# Downregulating *VAC14* in Guard Cells Causes Drought Hypersensitivity by Inhibiting Stomatal Closure

**DOI:** 10.3389/fpls.2020.602701

**Published:** 2020-12-17

**Authors:** Zong-Qi Wang, Qi Liu, Ju-Hua Wu, Juan Li, Jun-Min He, Yan Zhang, Sha Li

**Affiliations:** ^1^State Key Laboratory of Crop Biology, College of Life Sciences, Shandong Agricultural University, Tai'an, China; ^2^School of Life Sciences, Shaanxi Normal University, Xi'an, China

**Keywords:** ABA, drought, guard cell, PI(3, 5)P_2_, vacuole fission

## Abstract

Stomata are a key land plant innovation that permit the regulation of gaseous exchanges between the plant interior and the surrounding environment. By opening or closing, stomata regulate transpiration of water though the plant; and these actions are coordinated with acquisition of CO_2_ for photosynthesis. Stomatal movement is controlled by various environmental and physiological factors and associates with multiple intracellular activities, among which the dynamic remodeling of vacuoles plays a crucial role. Phosphatidylinositol 3,5-bisphosphate [PI(3,5)P_2_] is critical for dynamic remodeling of vacuoles. Its production requires a PI(3,5)P_2_-metabolizing complex consisting of FAB1/PIKfyve kinases, SAC phosphatases, and the scaffolding protein VAC14. Although genetic or pharmacological downregulation of PI(3,5)P_2_ causes hyposensitivity to ABA-induced stomatal closure, whether the effect of PI(3,5)P_2_ on stomatal movement is cell-autonomous and the physiological consequences of its reduction were unclear. We report that downregulating Arabidopsis *VAC14* specifically in guard cells by artificial microRNAs (amiR-VAC14) results in enlarged guard cells and hyposensitivity to ABA- and dark-induced stomatal closure. Vacuolar fission during stomatal closure is compromised by downregulating *VAC14* in guard cells. Exogenous application of PI(3,5)P_2_ rescued the amiR-VAC14 phenotype whereas PI(3,5)P_2_ inhibitor YM201636 caused wild-type plants to have inhibited stomatal closure. We further show that downregulating VAC14 specifically in guard cells impairs drought tolerance, suggestive of a key role of guard cell-produced PI(3,5)P_2_ in plant fitness.

## Introduction

Stomata are microscopic structures found on plant leaves which consist of two guard cells that surround a central pore. They are critical for plant responses to the environment (Hetherington, 2001; Kollist et al., 2014). Stomatal movement, i.e., opening or closure, determines the efficiency of water use and photosynthesis, as well as plant responses to abiotic and biotic stresses (Hetherington, 2001; Kollist et al., 2014). Stomatal movement can be regulated by environmental and physiological factors, including but not limited to illumination conditions, CO_2_ concentrations, humidity, and abscisic acid (ABA) (Hetherington, 2001; Kim et al., 2010; Kollist et al., 2014). ABA, darkness, low humidity, or high CO_2_ induce stomatal closure whereas light, high humidity, or low CO_2_ induce stomatal opening (Hetherington, 2001; Kim et al., 2010; Kollist et al., 2014).

Stomatal movements are associated with dynamic remodeling of vacuoles and changes of vacuolar acidity (Gao et al., 2005; Tanaka et al., 2007; Bak et al., 2013; Andres et al., 2014; Song et al., 2018). Vacuolar fission or convolution was observed during ABA-induced stomatal closure (Gao et al., 2005; Tanaka et al., 2007; Bak et al., 2013; Andres et al., 2014; Song et al., 2018) whereas vacuolar fusion or deconvolution was observed upon illumination (Gao et al., 2009) based on fluorescent labeling, pharmacological and genetic interference, as well as confocal imaging. The dynamic remodeling of vacuoles causes alterations in the turgor of guard cells, in which ion fluxes, especially potassium fluxes, are driving forces (Andres et al., 2014).

Phosphatidylinositol 3,5-bisphosphate [PI(3,5)P_2_] is critical for dynamic remodeling of vacuoles, not only in yeast and metazoans (McCartney et al., 2014) but also in plants (Bak et al., 2013; Andres et al., 2014; Novakova et al., 2014; Hirano et al., 2017a; Zhang et al., 2018). The level of PI(3,5)P_2_ is controlled by a class of FAB1/PIKfyve kinases and SAC phosphatases (van Leeuwen et al., 2004; McCartney et al., 2014). Homologs of FAB1/PIKfyve kinases (van Leeuwen et al., 2004; Whitley et al., 2009; Hirano et al., 2011, 2015; Serrazina et al., 2014) and those of SAC phosphatases (Zhong et al., 2005; Novakova et al., 2014; Zhang et al., 2018) have been identified and functionally characterized in plants. In addition to FAB1/PIKfyve kinases and SAC phosphatases whose activities antagonistically regulate PI(3,5)P_2_ production, VAC14, a scaffolding protein, interacts both with FAB1/PIKfyve kinases and SAC phosphatases to form a PI(3,5)P_2_-metabolizing complex and facilitate PI(3,5)P_2_ production (Takasuga and Sasaki, 2013; McCartney et al., 2014). The role of VAC14 is evolutionarily conserved since Arabidopsis VAC14 is also positively required for PI(3,5)P_2_ production, whose mutation resulted in male gametophytic lethality that is rescued by exogenous PI(3,5)P_2_ (Zhang et al., 2018).

The level of PI(3,5)P_2_ is low in plants (Meijer et al., 1999; Zonia and Munnik, 2004). However, hyperosmotic stresses rapidly induce its production (Meijer et al., 1999; Zonia and Munnik, 2004), suggesting a key role of PI(3,5)P_2_ in plant responses to stresses. Mutations of FAB1/PIKfyve kinases or the application of a PI(3,5)P_2_ production inhibitor YM201636 resulted in a reduced sensitivity to ABA-induced stomatal closure (Bak et al., 2013; Andres et al., 2014), suggesting a key role of PI(3,5)P_2_ in stomatal movement. However, it was not determined whether the effect of PI(3,5)P_2_ on stomatal movement is cell-autonomous or the physiological consequences of its reduction in guard cells.

Here, we report that downregulating Arabidopsis *VAC14* specifically in guard cells by artificial microRNAs (*Pro*_*GC*1_*:amiR-VAC14*) resulted in enlarged guard cells and hyposensitivity to ABA- and dark-induced stomatal closure. By examining vacuolar dynamics with fluorescence labeling, we determined that downregulating *VAC14* interfered with vacuolar fission during stomatal closure. Treatment with exogenous PI(3,5)P_2_ rescued the attenuated stomatal response caused by amiR-VAC14 whereas the application of PI(3,5)P_2_ inhibitor YM201636 on wild type mimicked ABA-hyposensitive stomatal closure. Although the *Pro*_*GC*1_*:amiR-VAC14* transgenic plants perform comparably to wild type under normal growth conditions, these plants are less tolerance to drought, suggestive of a key role of VAC14 in guard cells and in plant fitness.

## Materials and Methods

### Plant Materials and Growth Conditions

Arabidopsis Columbia-0 ecotype was used as the wild type. Transgenic plants including *Pro*_*UBQ*10_*:VAC14-GFP* (Zhang et al., 2018) and *Pro*_*UBQ*10_*:GFP-INT1* (Feng et al., 2017a,b) were described. Plant growth, transformation, and selection were performed as described (Zhou et al., 2013).

### RT-qPCRs

Total RNAs were extracted from abaxial epidermal peels of 4 WAG plants by using a Quick-RNA^TM^ MicroPrep Kit (ZYMO Research, #R1051), according to the manufacturer's instructions. Oligo(dT)-primed cDNAs were synthesized by using ReverTra Ace® qPCR RT Master Mix (TOYOBO, #FSQ-301). The RT-qPCRs were performed with the ABI QauntStudio^TM^ 6 Flex real-time system (Applied Biosystems, #4485697) using SYBR Green real-time PCR master mix (Toyobo) as described (Zhou et al., 2013). Primers used for *VAC14* are in Supplementary Table 1. Primers used for *GAPDH* were described (Zhou et al., 2013).

### Plasmid Construction

Primers used to generate amiR-VAC14 were designed based on the online tool WMD3 (http://wmd3.weigelworld.org/cgi-bin/webapp.cgi). Two independent amiR-VAC14 fragments were amplified with the primer pairs, ZP6117/ZP6118/ZP8745-ZP8748 for amiR1 or ZP6117/ZP6118/ZP8749-ZP8752 for amiR2. The resultant PCR fragments were inserted in pRS300 (Ossowski et al., 2008) to generate corresponding entry vectors. After enzymatic digestion with *Kpn*I/*Spe*I, the amiR fragments were used to replace the RFP-coding sequences in *Pro*_*GC*1_*:RFP* to generate two amiR plasmids *Pro*_*GC*1_*:amiR1-VAC14* and *Pro*_*GC*1_*:amiR2-VAC14*, respectively. Primers are in Supplementary Table 1.

### Measurement of Stomatal Aperture

For ABA-induced stomatal closure, the youngest fully expanded rosette leaves from plants at 4 WAG were floated on stomatal opening buffer containing 5 mM MES, 10 mM KCl, 50 μM CaCl_2_ (pH 6.15) for 3 hr in the light to pre-open stomata. The leaves were subsequently transferred to control buffer (0.1% ethanol, v/v), or buffers containing either 0, 10, or 100 μM ABA for 2.5 hr. Abaxial epidermal peels were taken for the measurement of stomatal apertures. For light-induced stomatal opening, leaves were excised from 4 WAG 12 hr-dark-adapted plants and incubated in the opening buffer (30 mM KCl, 10 mM MES-KOH pH6.5) for 0.5 h in the dark before the beginning of the light cycle. Leaves were illuminated for 0, 1, or 2 h before stomatal aperture measurement. For darkness-induced stomatal closure, leaves were excised from 4 WAG 12 hr-dark-adapted plants and incubated in the opening buffer for 3 h. Then the light-treated epidermal peels were transferred to darkness for 3 h before measurement.

### Quantification of Vacuoles

Guard cell vacuoles were counted from either GFP-INT1-labeled samples or Oregon Green (OG, Oregon Green carboxylic acid diacetate, Invitrogen) stained samples using ImageJ. For quantification, 30 pairs of guard cells for each genotype or treatment from three biological replicates were measured.

### Pharmacological Treatments

YM201636 (1 mM; #13576, Cayman Chemical) was prepared in DMSO. DMSO at the same dilution was added as the control. PI(3,5)P_2_ (#10008398, Cayman Chemical) was prepared in PBS buffer (pH 7.2). All experiments were repeated at least three times.

### Drought Assays

For drought treatment, five plants of each genotypes were grown in one pot and pots containing different genotypes are placed in the same tray. Plants were watered regularly. On 20 DAG, plants were watered to saturation (D0) and afterwards, no water was given to the plants till 35 DAG (D15). On 35 DAG, 150 mL water was given to every pot continuously for 3 days before images of the plants were taken (R3). For detached leaf assays, three fully expanded rosette leaves were, respectively, detached from plants and weighed immediately on aluminum foil. The detached leaves were then incubated on the foil on a laboratory bench and weighed at designated time intervals, as described (Jiang et al., 2012). Experiments were repeated three times. For thermal imaging, 4 WAG plants were imaged under 40–60% relative humidity for 30 min using an infrared camera, as described (Zheng et al., 2019).

### Fluorescence Microscopy

Fluorescence microscopy was performed by using a Zeiss LSM880 (Zeiss) confocal laser-scanning microscope with the following excitation/emission settings: 488 nm/505–550 nm for GFP or OG staining as described (Li et al., 2018; Song et al., 2018). Images were processed using Zeiss LSM image processing software (Zeiss) and Adobe Photoshop CS3 (Adobe). For quantification of VAC14-GFP intensity, abaxial epidermal peels were taken from 3 WAG plants and placed in stomatal opening buffer. Fluorescence intensity was calculated as average intensity in guard cells subtracted by that in leaf pavement cells using ImageJ.

### Accession Numbers

Sequence data in this article can be found in TAIR (The Arabidopsis Information Resource) under these accession numbers: At2g01690 for *VAC14*.

## Results

### Downregulating *VAC14* Results in Enlarged Guard Cells

**Figure 1:**
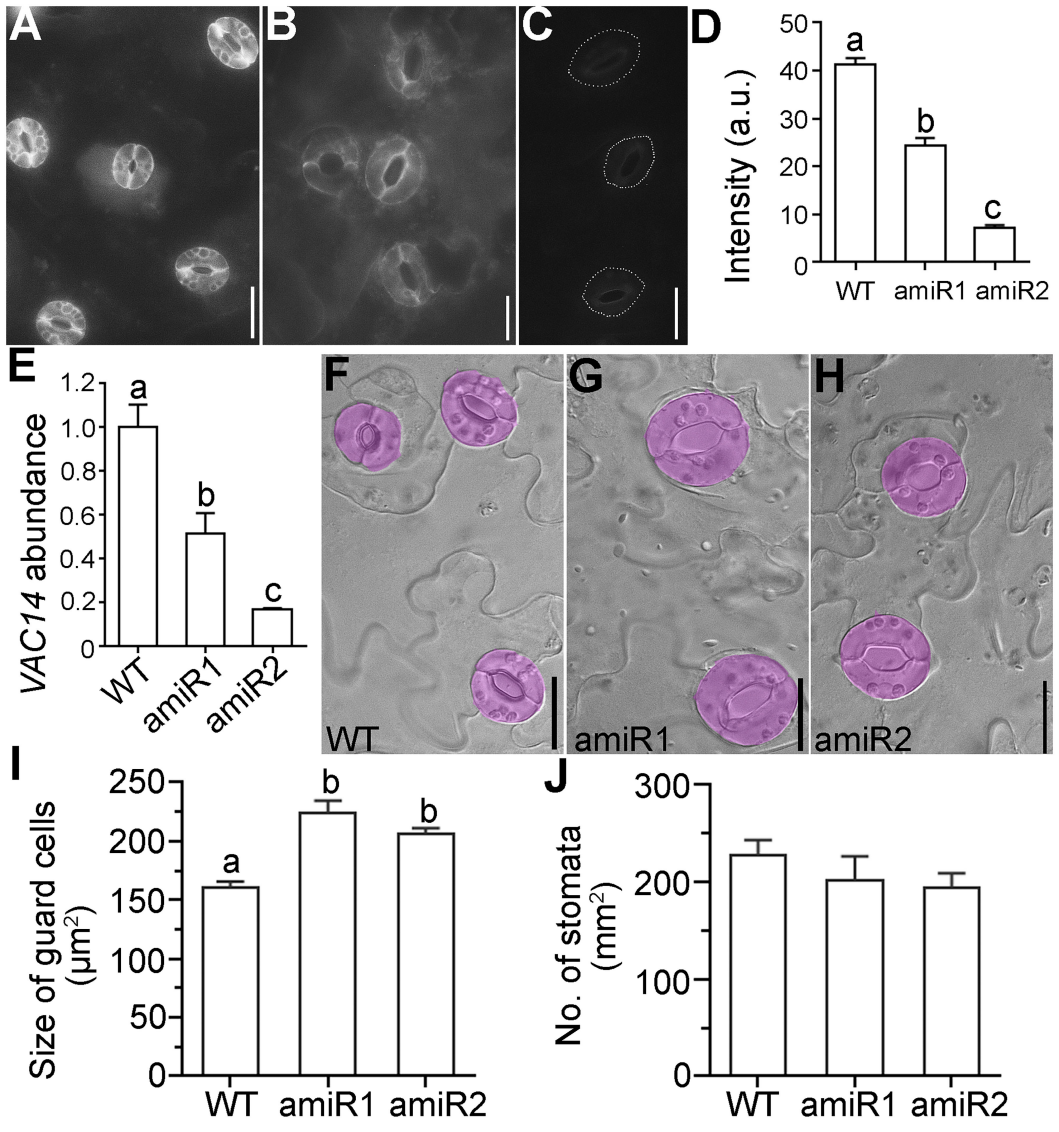
Downregulating *VAC14* results in enlarged guard cells. **(A–C)** Representative confocal laser scanning micrograph (CLSM) of abaxial epidermal peels from *Pro*_*UBQ*10_*:VAC14-GFP* (WT) **(A)**, *Pro*_*UBQ*10_*:VAC14-GFP;Pro*_*GC*1_*:amiR1-VAC14* **(B)**, or *Pro*_*UBQ*10_*:VAC14-GFP;Pro*_*GC*1_*:amiR2-VAC14* **(C)**. Dotted lines in **(C)** illustrate stomata pores because the GFP signals are too weak to be visible. **(D)** Intensity of VAC14-GFP in guard cells. a.u. indicates arbitrary unit. Values are means ± standard errors (SEM). Five plants of each genotype were used for fluorescence quantification. More than 60 guard cells from each plant were measured. **(E)** Relative *VAC14* transcript abundance in wild type, *Pro*_*GC*1_*:amiR1-VAC14*, or *Pro*_*GC*1_*:amiR2-VAC14* by RT-qPCRs. RNAs were extracted from leaf epidermal peels. Values are means ± SEM (*n* = 3). **(F–H)** Representative DIC images of leaf epidermal from wild type **(F)**, *Pro*_*GC*1_*:amiR1-VAC14* **(G)**, or *Pro*_*GC*1_*:amiR2-VAC14*. **(H)** Guard cells are pseudo-colored in lilac. **(I,J)** Quantification of guard cell size **(I)** or density **(J)**. Values are means ± SEM (*n* = 5). For guard cell size, 150 stomata from 5 leaves of each genetic background were measured. For stomata density, 15 images taken from 5 leaves of each genetic background were measured. Different letters in **(D,E,I)** indicate significantly different groups (OneWay ANOVA, Tukey's multiple comparisons test, *P* < 0.05). No significant difference was detected among three genotypes in **(J)** (OneWay ANOVA, Tukey's multiple comparisons test, *P* > 0.05). Bars = 20 μm.

We previously demonstrated that Arabidopsis VAC14 is a key protein for PI(3,5)P_2_ production, whose mutation resulted in male gametophytic lethality (Zhang et al., 2018). Although *VAC14* is expressed in various tissues and developmental stages, its expression in mature guard cells is quite high (Zhang et al., 2018). Because vacuolar dynamics play a key role in stomatal movement (Gao et al., 2005; Tanaka et al., 2007; Andres et al., 2014; Song et al., 2018) and PI(3,5)P_2_ mediates vacuolar acidification and endomembrane dynamics in plants (Hirano et al., 2011, 2015, 2017a; Novakova et al., 2014), we considered the possibility that VAC14 plays a role in stomatal movement. To test this hypothesis, we generated artificial microRNAs to downregulate *VAC14* specifically in guard cells by using *Pro*_*GC*1_ that is specific for guard cells (Yang et al., 2008; Song et al., 2018). We generated two amiR-VAC14 constructs, *Pro*_*GC*1_*:amiR1-VAC14* and *Pro*_*GC*1_*:amiR2-VAC14*, which targeted to different regions of the *VAC14* sequence. We first verified the activity of amiR1 and amiR2 by introducing the transgenes into *Pro*_*UBQ*10_*:VAC14-GFP* transgenic plants, previously used in the complementation of *VAC14* loss-of-function mutants (Zhang et al., 2018). Based on fluorescence intensity of guard cells from leaf abaxial epidermal peels, we concluded that both amiR transgenes are functional (Figures 1A–D). To confirm the results, we also performed quantitative real-time PCRs (RT-qPCRs) to examine the transcript abundance of *VAC14*. Although *VAC14* is constitutively expressed whereas amiR-VAC14 is only active in mature guard cells, we were able to detect a significant reduction of *VAC14* abundance by both transgenes in leaf abaxial epidermal peels (Figure 1E), demonstrating the identity of the *Pro*_*GC*1_*:amiR-VAC14* transgenic plants.

Both *Pro*_*GC*1_*:amiR1-VAC14* and *Pro*_*GC*1_*:amiR2-VAC14* transgenic plants were comparable to wild type during vegetative (Supplementary Figure 1A) and reproductive growth (Supplementary Figure 1B), consistent with the guard cell-specific expression of transgenes. Stomatal density of guard cells was not affected (Figure 1J), as would be expected since amiR-VAC14 was only active in mature guard cells. However, guard cells of amiR-VAC14 plants were significantly larger than those of wild type (Figures 1F–I), indicating that downregulation *VAC14* in guard cells promoted their growth.

### amiR-VAC14 Plants Are Hyposensitive to ABA- and Dark-Induced Stomatal Closure

**Figure 2:**
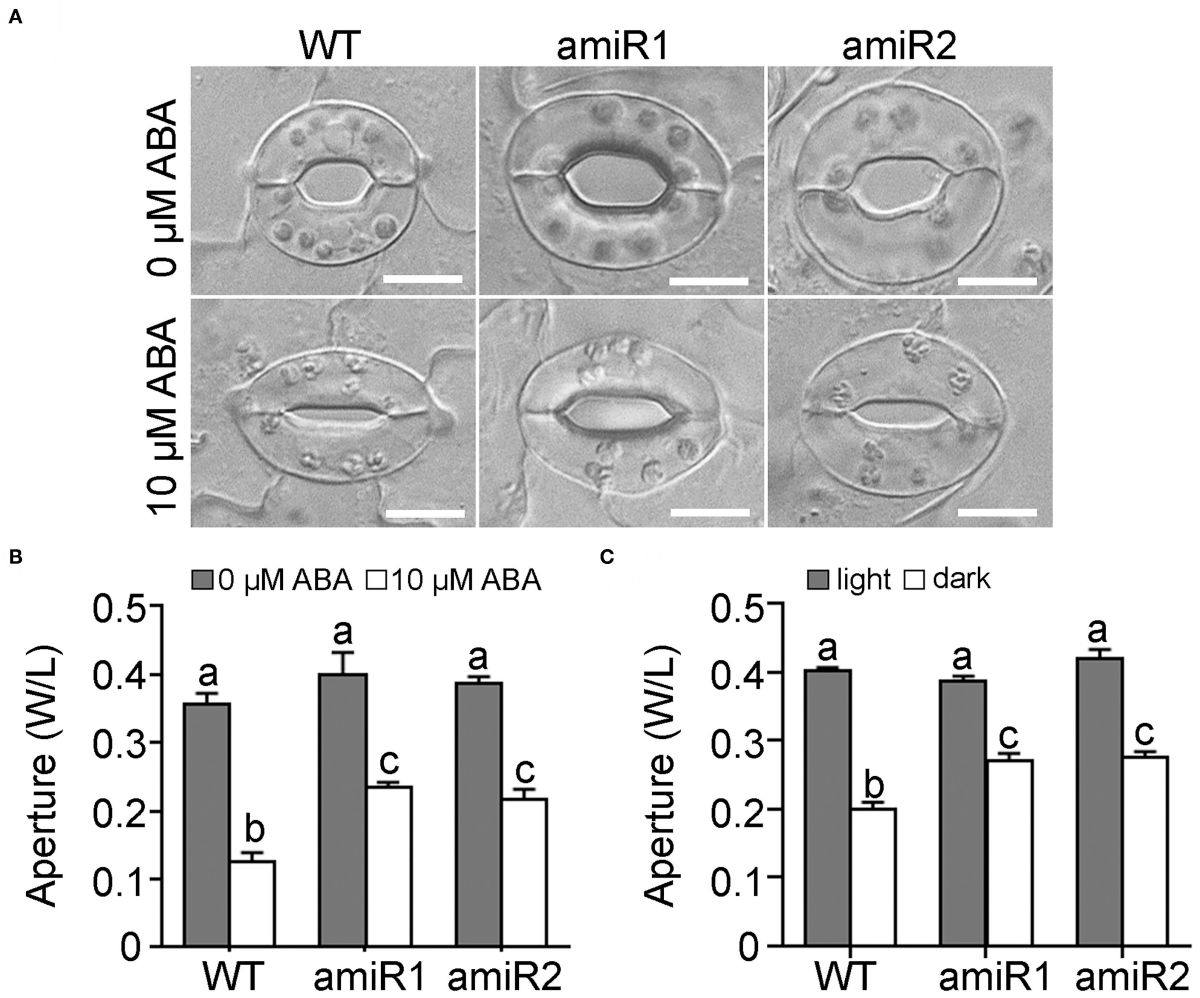
Downregulating *VAC14* in guard cells results in reduced stomatal closure by ABA or dark. **(A,B)** Representative DIC images **(A)** or quantification **(B)** of wild type, *Pro*_*GC*1_*:amiR1-VAC14*, or *Pro*_*GC*1_*:amiR2-VAC14* stomata for ABA-induced stomatal closure. Rosette leaves with preopened stomata were treated with 0 or 10 μM ABA for 2 h before the apertures were measured. **(C)** Stomata aperture upon darkness. Results in **(B,C)** are means ± SE (*n* = 3). Each experiment includes 30 stomata from three epidermal peels. Different letters indicate significantly different groups (Two-Way ANOVA, Tukey's multiple comparisons test, *P* < 0.05). Bars = 10 μm.

To examine the possible effect of amiR-VAC14 on stomatal movement, we first examined stomatal movement in response to ABA. Because the increased size of amiR-VAC14 guard cells, we used the ratio of width to length (W/L) to define stomatal aperture, as described (Shang et al., 2016). Stomatal closure was induced upon leaf epidermal peels being incubated at 10 μM ABA for 2.5 h (Figures 2A,B), as reported (Song et al., 2018). Although stomata of amiR-VAC14 were responsive to ABA, aperture of stomata was significantly larger than that of wild type (Figures 2A,B), suggesting ABA hyposensitivity. To determine whether amiR-VAC14 caused hyposensitivity specifically to ABA or to stomatal closure in general, we tested the effect of darkness since it also induces stomatal closure (Isner et al., 2017; Zhang et al., 2017). The same was true for darkness-induced stomatal closure, i.e., downregulating *VAC14* in guard cells reduced the sensitivity of stomatal movement in response to darkness (Figure 2C). We also quantified stomatal pore areas in response to ABA treatment. Indeed, amiR-VAC14 resulted in hyposensitivity to ABA-induced stomatal closure (Supplementary Figure 2). Next, we examined the response of *Pro*_*GC*1_*:amiR-VAC14* stomata to light, which induces stomatal opening (Song et al., 2018). We determined that amiR-VAC14 stomata showed a slower response to illumination than those of wild type (Supplementary Figure 3). This was confirmed also by quantifying stomata pore areas upon light treatment (Supplementary Figure 2). However, the slow response of amiR-VAC14 stomata to light-induced opening was more likely due to the fact that stomata from 12 h-dark-adapted amiR-VAC14 plants did not close as well as those of wild type (Supplementary Figure 3). Illumination for 2 h induced stomata of amiR-VAC14 to reach the maximum aperture comparably to those of wild type (Supplementary Figure 3). These results suggested that downregulating *VAC14* in guard cells compromised the ability of stomata to close but not opening.

### Vacuolar Fission During Stomatal Closure Is Compromised by amiR-VAC14

**Figure 3:**
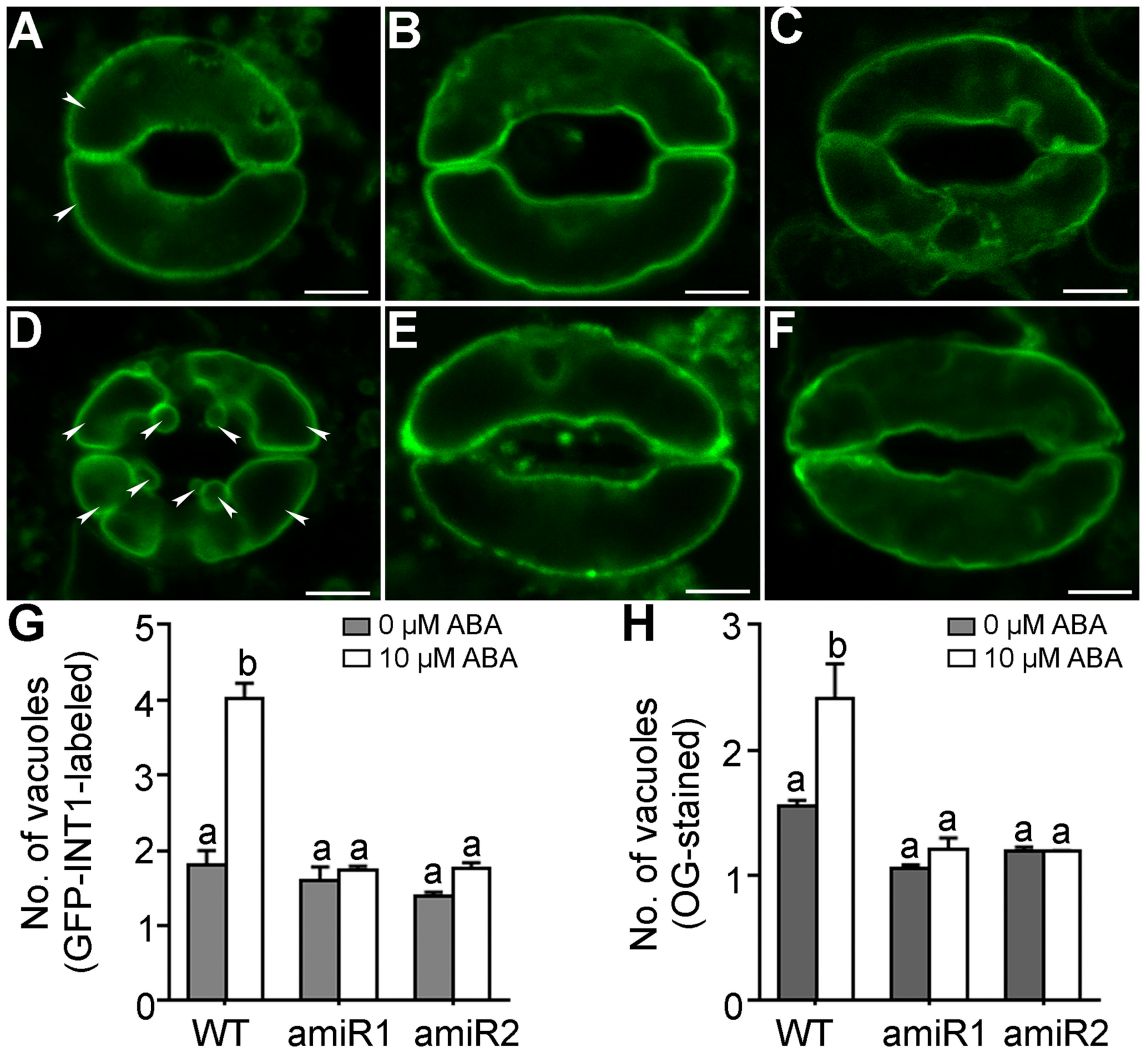
Vacuolar fission during stomatal closure is compromised by *amiR-VAC14*. **(A–F)** Representative CLSM of a guard cell from *Pro*_*UBQ*10_*:GFP-INT1* (WT), *Pro*_*UBQ*10_*:GFP-INT1;Pro*_*GC*1_*:amiR1-VAC14*, or *Pro*_*UBQ*10_*:GFP-INT1;Pro*_*GC*1_*:amiR2-VAC14*, upon 0 μM **(A–C)** or 10 μM ABA **(D–F)** treatment for 2.5 h. Arrowheads point at separately vacuoles in wild-type guard cells. **(G,H)** Number of vacuoles/guard cell by GFP-INT1-labeling **(G)** or by OG-staining **(H)**. Results are means ± SE (*n* = 3). Each experiment includes 30 stomata from three epidermal peels. Different letters indicate significantly different groups (Two-Way ANOVA, Tukey's multiple comparisons test, *P* < 0.05). Bars = 5 μm.

*VAC14* loss-of-function compromised vacuolar fission during pollen development in Arabidopsis (Zhang et al., 2018). We therefore hypothesized that amiR-VAC14 affected stomatal closure by impairing vacuolar fission, which is critical for stomatal closure (Gao et al., 2005; Andres et al., 2014). To test this idea, we introduced *Pro*_*GC*1_*:amiR-VAC14* into *Pro*_*UBQ*10_:*GFP-INT1* (Feng et al., 2017a,b), in which GFP-INT1 labels the tonoplast. In wild type, ABA treatment of 10 μM ABA for 2.5 hrs induced vacuolar fission (Figures 3A,D), from an average of two vacuoles per guard cell to around four (Figure 3G). In comparison, the same ABA treatment hardly affected the numbers of vacuoles in amiR-VAC14 (Figures 3B–G), indicating the failure of vacuolar fission. To confirm this result, we also stained guard cells with Oregon Green (OG), a fluorescence dye specific for vacuoles (Andres et al., 2014; Zhang et al., 2018). By CLSM projections of Z-stack images, we confirmed that ABA treatment hardly induced vacuolar fission in amiR-VAC14 guard cells, in great contrast to that in wild type (Figure 3H and Supplementary Figure 4). On the other hand, stomatal opening accompanies vacuolar fusion (Gao et al., 2009; Andres et al., 2014). Two hours of illumination induced vacuolar fusion from average 4 vacuoles to 2 vacuoles per guard cell in wild type (Supplementary Figure 5). However, the same illumination did not cause a significant change in the number of vacuoles in amiR-VAC14 guard cells (Supplementary Figure 5), suggesting that vacuolar dynamics in general are affected by amiR-VAC14.

### Exogenous PI(3,5)P_2_ Rescued While PI(3,5)P_2_ Inhibitor Mimicked amiR-VAC14 in Defective Stomatal Closure

**Figure 4:**
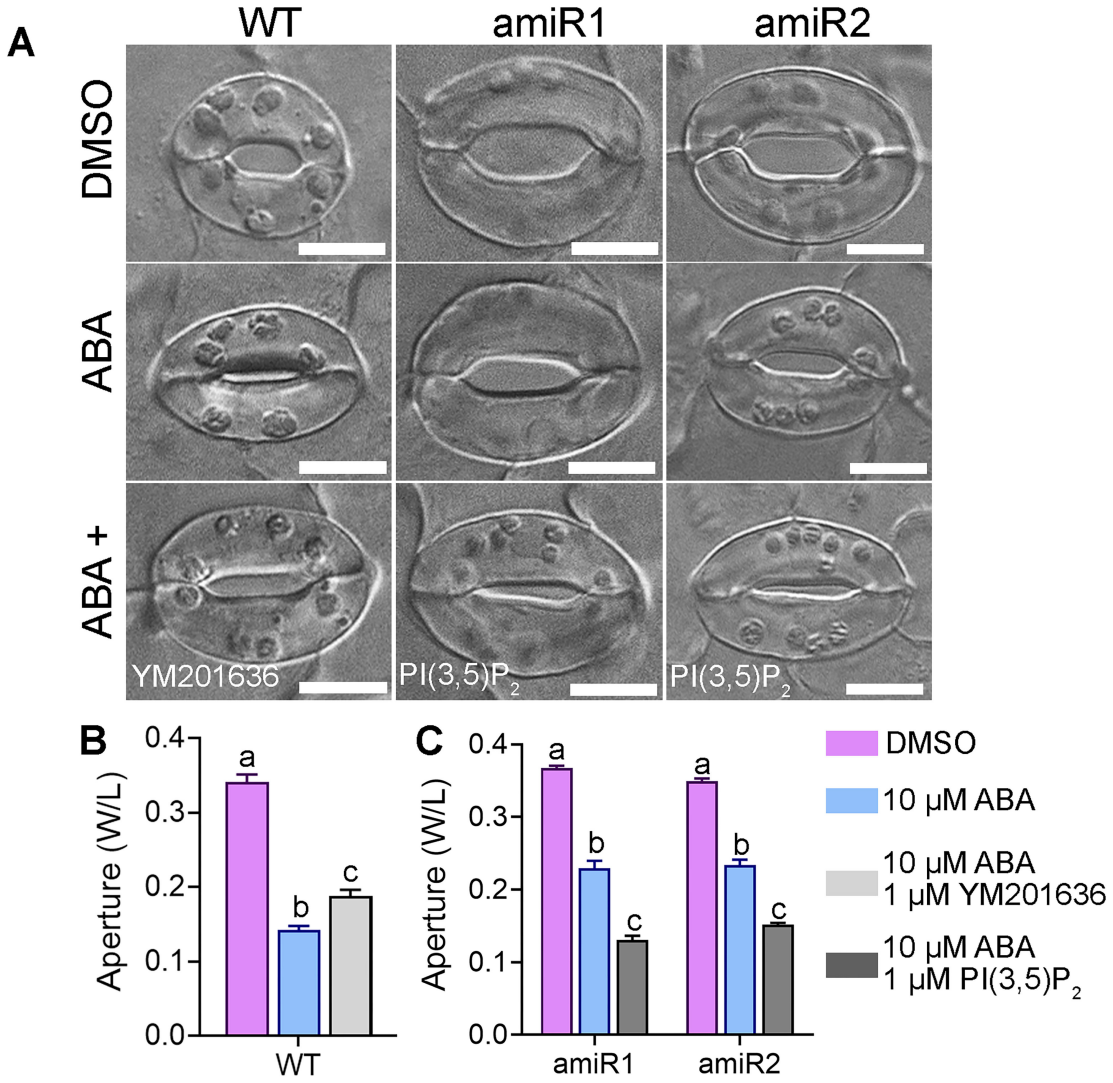
Exogenous PI(3,5)P_2_ rescued while PI(3,5)P_2_ inhibitor mimicked ABA-hyposensitive stomatal closure of VAC14-amiR. **(A)** Representative DIC images of wild-type, *Pro*_*GC*1_*:amiR1-VAC14*, or *Pro*_*GC*1_*:amiR2-VAC14* stomata. Rosette leaves with preopened stomata were treated with 0 μM ABA (DMSO), 10 μM ABA, 10 μM ABA/1 μM YM201636, or 10 μM ABA/1 μM PI(3,5)P_2_ treatment for 2 h before the apertures were measured. **(B,C)** Stomata aperture. Results are means ± SE (*n* = 3). Each experiment includes 30 stomata from three epidermal peels. Different letters indicate significantly different groups (Two-Way ANOVA, Tukey's multiple comparisons test, *P* < 0.05). Bars = 10 μm.

VAC14 is important for PI(3,5)P_2_ production both in yeast (Takasuga and Sasaki, 2013; McCartney et al., 2014) and in Arabidopsis while vacuolar dynamics are regulated by PI(3,5)P_2_ (Bak et al., 2013; Andres et al., 2014; Zhang et al., 2018). To verify that the hyposensitive stomatal closure of amiR-VAC14 plants was due to a reduced PI(3,5)P_2_ level, we used a pharmacological approach. YM201636 inhibits the production of PI(3,5)P_2_ in yeast and metazoan cells (Jefferies et al., 2008), as well as in plants (Hirano et al., 2017a,b; Zhang et al., 2018). Indeed, the application of 1 μM YM201636 (Bak et al., 2013; Zhang et al., 2018) significantly reduced ABA-mediated stomatal closure (Figures 4A,B), suggesting that PI(3,5)P_2_ positively regulates ABA signaling in guard cells. On the other hand, application of 1 μM PI(3,5)P_2_ rescued the hyposensitive response of amiR-VAC14 stomata to ABA (Figures 4A,C), rendered it comparable to wild type (Figures 4A,B). Exogenous PI(3,5)P_2_ rescued while the PI(3,5)P_2_ inhibitor mimicked amiR-VAC14 in defective stomatal closure, confirming that the effect of amiR-VAC14 on stomatal closure is due to a reduction of PI(3,5)P_2_.

### amiR-VAC14 Is Less Tolerance to Drought

**Figure 5:**
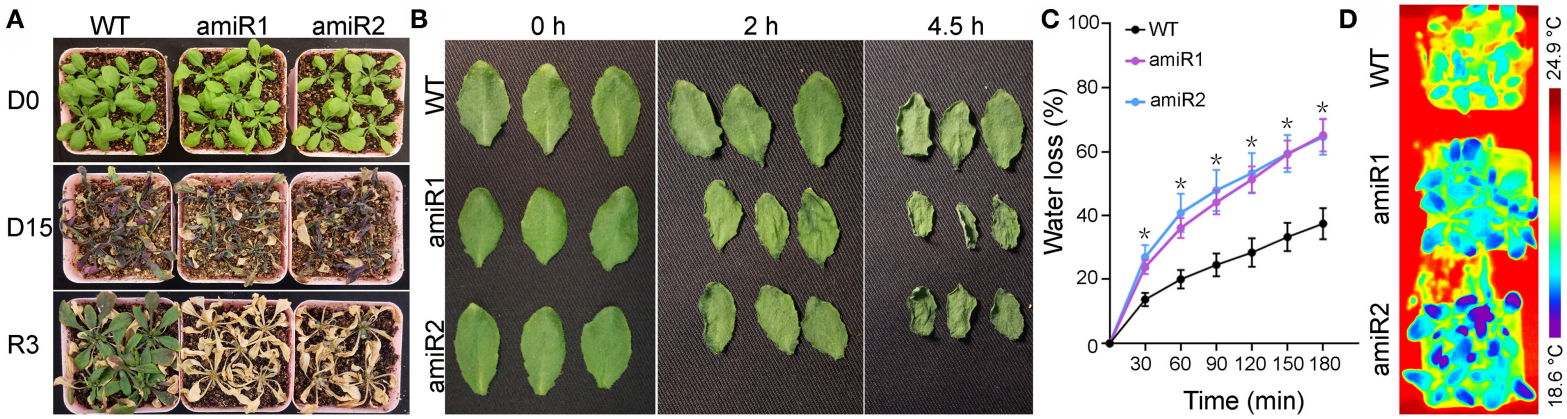
Downregulating *VAC14* in guard cells reduces drought tolerance. **(A)** A representative drought experiment with wild type, *Pro*_*GC*1_*:amiR1-VAC14*, or *Pro*_*GC*1_*:amiR2-VAC14*. Plants at 20 DAG (D0) were stopped watering for 15 days (D15) and then rewatered for 3 days (R3). **(B)** A representative detached leaf assay with wild type, *Pro*_*GC*1_*:amiR1-VAC14*, or *Pro*_*GC*1_*:amiR2-VAC14*. **(C)** Cumulative leaf transpiration water loss from detached rosette leaves of wild type, *Pro*_*GC*1_*:amiR1-VAC14*, or *Pro*_*GC*1_*:amiR2-VAC14*. Results shown are means ± SE of three replicates. Asterisks indicate significant difference from wild type at the same time point (OneWay ANOVA, Tukey's multiple comparisons test, *P* < 0.05). **(D)** Infrared thermography of wild type, *Pro*_*GC*1_*:amiR1-VAC14*, or *Pro*_*GC*1_*:amiR2-VAC14*. Well-watered 3 WAG plants were used. The colors of leaves represent leaf temperature.

Because stomatal movement is critical for water transpiration, especially under drought, the physiological consequences of amiR-VAC14 would be reduced drought tolerance. To test this hypothesis, we performed the following assays. Wild-type and amiR-VAC14 plants were grew under long day condition for 20 days after germination (DAG) with regular watering. Starting from 20 DAG (D0), water was withheld from plants for 15 days (D15). After that, regular watering at equal quantity was resumed for 3 days (R3). Drought for 15 days caused leaf necrosis and withering (Figure 5A), as described (Song et al., 2018). Wild-type plants rapidly recovered upon 3 days of rewatering (Figure 5A), as described (Takahashi et al., 2018). By contrast, amiR-VAC14 plants did not recover and finally died (Figure 5A). Next, we performed a water loss experiment by a detached leaf assay (Jiang et al., 2012). Indeed, detached leaves from amiR-VAC14 withered in a much faster way than those of wild type (Figures 5B,C), indicating higher transpiration water loss. Because stomatal density was not affected by amiR-VAC14 (Figure 1), the wilty phenotype was due to compromised stomatal closure. Finally, we surveyed leaf thermal profiles of wild-type and amiR-VAC14 plants using an infrared thermal imaging camera (Zheng et al., 2019). The leaf thermal profile of amiR-VAC14 exhibited a cooler phenotype than that of wild type (Figure 5D). These results also showed a hypersensitivity of amiR-VAC14 to drought.

## Discussion

In this study, we demonstrate that down-regulation of *VAC14* via artificial microRNA reduced PI(3,5)P_2_ production in guard cells and thereby compromised stomatal movement in response to ABA or dark treatment (Figure 2). Although stomatal opening of amiR-VAC14 plants was less sensitive to light (Supplementary Figure 3), the effect might have been caused by the larger aperture of amiR-VAC14 stomata after dark-adaptation.

It was reported that reducing PI(3,5)P_2_ levels either genetically or pharmacologically impairs vacuolar fragmentation (Bak et al., 2013; Novakova et al., 2014; Hirano et al., 2017a; Zhang et al., 2018). Functional loss of Arabidopsis *VAC14* resulted in defective vacuolar fragmentation at pollen mitosis I during pollen development (Zhang et al., 2018), supporting a positive role of PI(3,5)P_2_ in promoting vacuolar fragmentation. We also show here that vacuolar fragmentation in guard cells during stomatal closure was completed inhibited by amiR-VAC14 (Figure 3, Supplementary Figure 4). On the other hand, reducing PI(3,5)P_2_ levels by YM201636 was reported to have caused smaller vacuoles in cells at root tips (Hirano et al., 2017a). A possible explanation for the seemingly discrepant results is that PI(3,5)P_2_ may influence vacuolar dynamics in a cell-specific manner. Cells at the root tips are mostly meristem cells that are actively dividing whereas guard cells are fully differentiated. Strikingly, stomata of amiR-VAC14 did close (Figure 2) without vacuolar fragmentation in guard cells (Figure 3). These results suggest that although vacuolar fragmentation is always associated with stomatal closure, other intracellular activities, such as dynamic organization of actin microfilaments (Gao et al., 2009; Jiang et al., 2012; Zheng et al., 2019), may be enhanced to compensate for its absence, leading to stomatal closure.

Downregulating *VAC14* or reducing PI(3,5)P_2_ production in guard cells compromised drought tolerance of Arabidopsis (Figure 5) without affecting plant growth under optimal growth conditions (Supplementary Figure 1). It is thus a tempting possibility that enhancing PI(3,5)P_2_ production in guard cells generates more drought-tolerant plants to adapt to environmental changes in the future. However, constitutive overexpression of PI(3,5)P_2_-generating kinase FAB1 resulted in pleiotropic developmental defects (Hirano et al., 2011, 2017a). In fact, *FAB1* gain-of-function caused pollen abortion (Hirano et al., 2011) similar to that caused by *FAB1* loss of function (Whitley et al., 2009) or *VAC14* loss of function (Zhang et al., 2018). These results imply the importance of a fine-tuned PI(3,5)P_2_ level. Indeed, the production of PI(3,5)P_2_ is enhanced by hyperosmotic stresses (Meijer et al., 1999; Zonia and Munnik, 2004). Although it is unclear which component of the PI(3,5)P_2_-synthesizing complex is activated by hyperosmotic stresses, the result indicates the necessity of a dynamic regulation of PI(3,5)P_2_ levels.

Studies in yeast indicated that PI(3,5)P_2_ controls vacuolar fission by controlling vacuolar ATPases to regulate vacuolar acidification (Michell et al., 2006; Li et al., 2014). In plants, YM201636 inhibits vacuolar acidification in Arabidopsis while mutations of vacuolar ATPases caused insensitivity to ABA-induced stomatal closure (Bak et al., 2013), suggesting a role of PI(3,5)P_2_ in the regulation of vacuolar ATPases. However, a recent study showed that PI(3,5)P_2_ inhibits an inwardly rectifying channel rather than affects the activity of proton pumps on Arabidopsis vacuoles (Carpaneto et al., 2017). The conductance is proposed to be mediated by chloride channel a (CLC-a) (Carpaneto et al., 2017), an anion/H^+^ exchanger whose functional loss causes hyposensitivity to light-induced stomatal opening as well as ABA-induced stomatal closure (Wege et al., 2014). Based on these studies, a reduced PI(3,5)P_2_, i.e. increased CLC-a activity, would cause hypersensitivity to both light-induced stomatal opening as well as ABA-induced stomatal closure. By contrast, we demonstrated that reduced PI(3,5)P_2_ levels, either by amiR-VAC14 or by YM201636 treatment, resulted in reduced sensitivity to ABA-induced stomatal closure (Figures 2, 4) and light-induced stomatal opening (Supplementary Figure 3). The discrepancy will need to be solved in the future.

PI(3,5)P_2_ controls vacuolar fission also by regulating protein sorting at the multivesicular bodies (MVB) in yeast (Takasuga and Sasaki, 2013). By using a fluorescence probe for PI(3,5)P_2_, the lipid was found to be associated with the prevacuolar compartments (PVC/MVB) in plants (Hirano et al., 2017b) and mediates protein sorting among different endomembrane compartments (Hirano et al., 2017a), suggesting a similar regulatory mechanism.

## Data Availability Statement

The original contributions presented in the study are included in the article/Supplementary Materials, further inquiries can be directed to the corresponding author/s.

## Author Contributions

Z-QW and QL performed the experiments with the assistance of J-HW and JL. SL, YZ, Z-QW, and J-MH analyzed the data. SL and YZ wrote the article with the input of Z-QW. All authors contributed to the article and approved the submitted version.

## Conflict of Interest

The authors declare that the research was conducted in the absence of any commercial or financial relationships that could be construed as a potential conflict of interest.
