# Supplementary material for: Downregulating VAC14 in Guard Cells Causes Drought Hypersensitivity by Inhibiting Stomatal Closure
Source: Front Plant Sci. 2020 Dec 17;11:602701. doi: 10.3389/fpls.2020.602701 (PMC7773697; doi:10.3389/fpls.2020.602701)
Supplement: Supplementary file 1 [file Image_1.PDF]

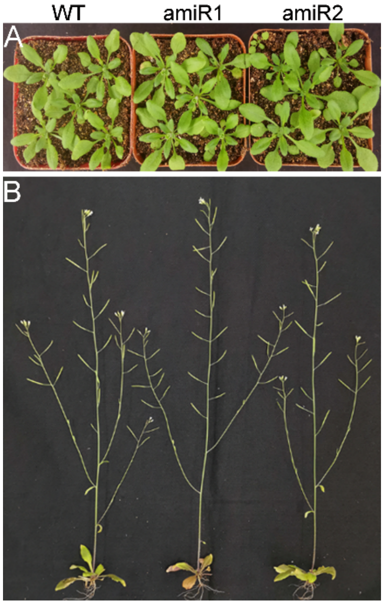

Figure S1. The expression of amiR-VAC14 in guard cells did not affect plant growth. (A-B) Representative wild-type (WT), *Pro$_{GC1}$:amiR1-VAC14* (amiR1), or *Pro$_{GC1}$:amiR2-VAC14* plants (amiR2) at 3 weeks after germination (WAG) (A) or 5 WAG (B).

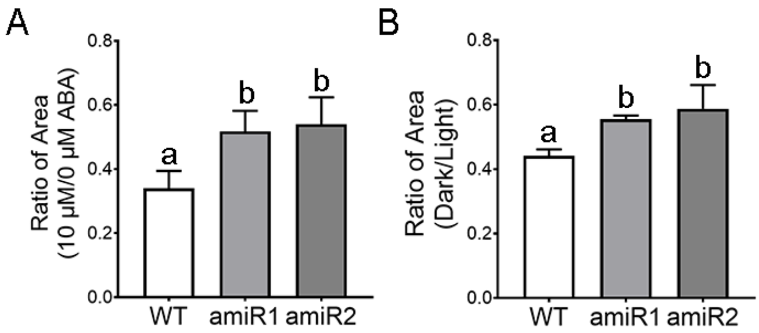

Figure S2. Downregulating *VAC14* in guard cells results in reduced stomatal closure by ABA or dark.

(A) Ratio of stomata pore area upon 10 µM ABA treatment versus 0 µM ABA treatment (A). (B) Ratio of stomata pore area upon dark versus. Results are means ± SE (n = 3). Each experiment includes 30 stomata from three epidermal peels. Different letters indicate significantly different groups (OneWay ANOVA, Tukey's multiple comparisons test, $P<0.05$).

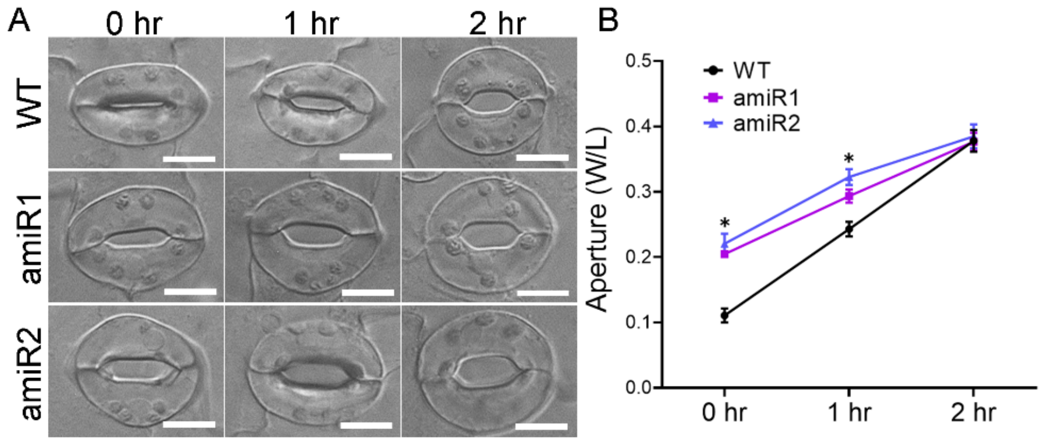

Figure S3. Downregulating *VAC14* in guard cells results in slow responses to light.
(A-B) Representative DIC images (A) or quantification (B) of light-induced stomatal opening in the wild type, *Pro$_{GC1}$:amiR1-VAC14*, or *Pro$_{GC1}$:amiR2-VAC14*. Rosette leaves with preclosed stomata were illuminated for designated time points before the apertures were measured. Results are means ± SE (n = 3). Each experiment includes 30 stomata from three epidermal peels. Asterisks indicate that two amiR lines show significant difference from wild type in corresponding time points (OneWay ANOVA, Tukey's multiple comparisons test, $P<0.05$). Bars = 10 µm.

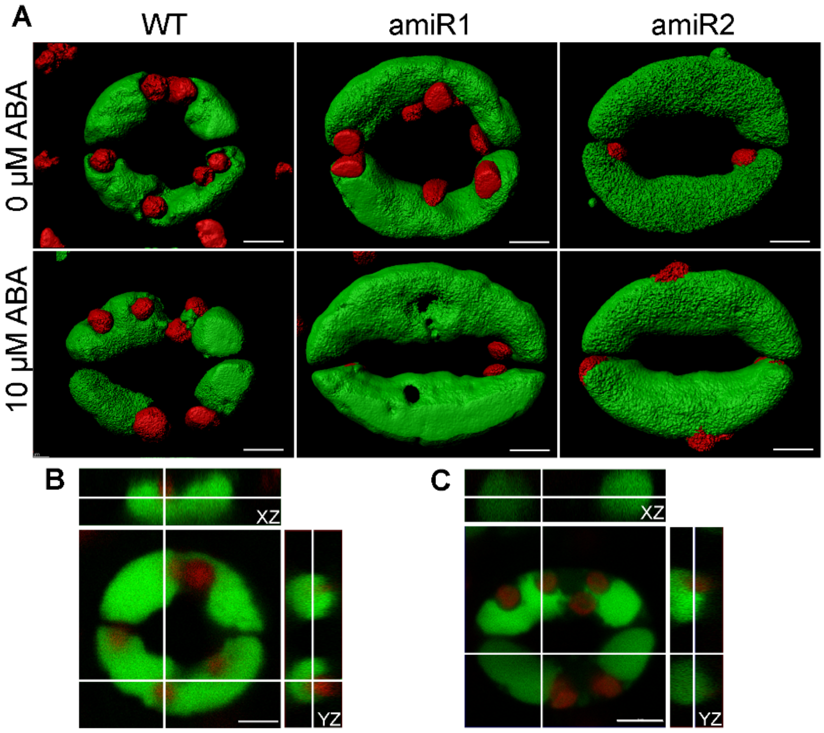

Figure S4. Vacuolar fission during stomatal closure is compromised by amiR-VAC14. (A) Representative 3D surface rendering of an OG-stained guard cell from wild type, *Pro$_{GC1}$:amiR1-VAC14*, or *Pro$_{GC1}$:amiR2-VAC14* upon 0 µM or 10 µM ABA treatment for 2.5 hrs. Auto-fluorescence of chloroplasts is shown in red. (B-C) Orthogonal views of wild-type 3D surface rendering images upon 0 µM ABA (B) or 10 µM ABA (C) treatments. XZ indicates the parallel projection of XZ plane whereas YZ indicates the parallel projection of YZ plane. The XZ plane and YZ plane highlighted with white lines are orthogonal. Bars = 5 µm.

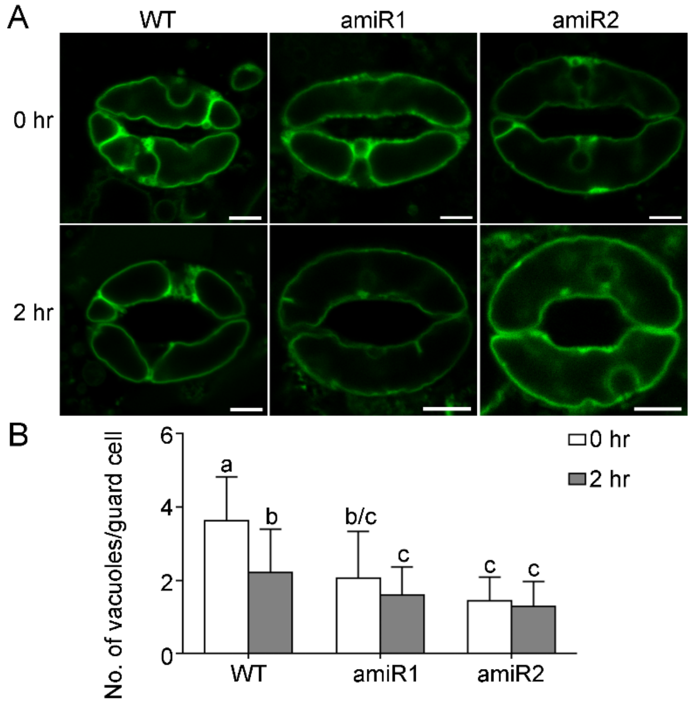

Figure S5. Vacuolar fusion during stomata opening in amiR-VAC14 plants is insensitive to light.

(A) Representative CLSM of a guard cell from *Pro_{UBQ10}:GFP-INT1* (WT), *Pro_{UBQ10}:GFP-INT1;Pro_{GC1}:amiR1-VAC14*, or *Pro_{UBQ10}:GFP-INT1;Pro_{GC1}:amiR2-VAC14*, in darkness (0 hr) or upon 2 hr illumination. (B) Number of vacuoles/guard cell by GFP-INT1-labeling. Rosette leaves with preclosed stomata were illuminated for designated time points before the number of vacuoles was measured. Results are means ± SD (n = 3). Each experiment includes 30 stomata from three epidermal peels. Different letters indicate significantly different groups (Two-Way ANOVA, Tukey's multiple comparisons test, $P<0.05$). Bars = 5 µm.

Table S1. Oligos used in this study.

Application	No.	Sequence 5'-3'
Cloning of amiR-VAC14	ZP6117	CTGCAAGGCGATTAAGTTGGGTAAC
	ZP6118	GCGGATAACAATTTCACACAGGAAACAG
	ZP8745	GATGTTAAAGCTGTAATTGACCATCTCTCTTTTGTATTCC
	ZP8746	GATGGTCAATTACAGCTTTAACATCAAAGAGAATCAATGA
	ZP8747	GATGATCAATTACAGGTTTAACTTCACAGGTCGTGATATG
	ZP8748	GAAGTTAAACCTGTAATTGATCATCTACATATATATTCCT
	ZP8749	GATGTCAGCATAATAACGCACGGTCTCTCTTTTGTATTCC
	ZP8750	GACCGTGCGTTATTATGCTGACATCAAAGAGAATCAATGA
	ZP8751	GACCATGCGTTATTAAGCTGACTTCACAGGTCGTGATATG
	ZP8752	GAAGTCAGCTTAATAACGCATGGTCTACATATATATTCCT
RT-qPCR for *VAC14*	ZP4063	GAATCTACATCGTGGCCAGGC
	ZP3535	AAGCTGTAATTGACCAGGGCC
